# Is Homœopathy Sufficient in All Cases

**Published:** 1889-02

**Authors:** W. G. Brownell

**Affiliations:** Chairman, 122 North Avenue; Rochester, N. Y.


### IS HOMŒOPATHY SUFFICIENT IN ALL CASES.

ROCHESTER, N. Y., December 24th, 1888.

DEAR DOCTOR:—As chairman of a Committee appointed by the President of the Rochester Hahnemannian Society, to prepare a paper for publication on the theme, "Is the homœopathic remedy always sufficient to relieve suffering in incurable cases?" I come to you for information. Your name has been suggested as one who must have record of cases in which euthanasia has been produced by the simillimum, and as one who never resorts to allopathic measures for palliation.

The Committee referred to above is composed of Julius G. Schmitt, M. D., W. H. Baker, M. D., and myself. We believe that the question can be answered affirmatively, and we have some evidence to that effect, but more cases of all the classes of incurable maladies that have been relieved by the simillimum are desired, notably such serious cases as the different cancerous diseases of the uterus, and diseases of the kidneys and lungs. Of course, it is evident that cases in which a post-mortem examination has followed such treatment are especially desirable.

We feel encouraged to ask you for such evidence or facts, knowing through your utterances published in our journals and the *Transactions of the I. H. A.*, of your intense love for the truth in Homœopathy. You are, no doubt, aware that the Rochester Hahnemannian Society has taken such a step recently as to precipitate a crisis here. Many laymen are inquiring as to the division thus established, and some of our eclectics, calling themselves homœopaths, have publicly stated through the medium of the daily papers that the only difference between the two factions is that they "employ more powerful measures to relieve cases sick beyond recovery," thus, as you will readily see, "begging the (real) question." Our position has been taken after much thought, and to advance the interests of pure Homœopathy, and consequently we cannot shirk any of the responsibilities following such a move. The publication of an affirmative answer to the question has become imperative. We desire that the answer shall be a most emphatic one, and backed by the most positive evidence, so we are placing ourselves in communication with all the representative Hahnemannians in the United States.

Will you kindly send, as soon as possible, any cases you may have that you consider important; also a statement of your ordinary method of handling incurable cases; may we use your name in connection with such treatment? We inclose a circular letter, recently published, which will state our position to you more fully.

Fraternally yours,

W. G. Brownell, M. D.,
*Chairman*,
122 North Avenue.

---

Our Answer: In reply to the above circular, we would briefly say that in our experience Homœopathy strictly practiced, is capable of giving the fullest peace, comfort, and rest to the incurable patient, whether it be a case of cancer, of tuberculosis, or what not. Secondly, that in *all* curable diseases, Hahnemannian Homœopathy cures most speedily and effectively.

The method of applying the law of similars is always the same, whether the case be curable or incurable. Prescribe carefully for the symptoms of the *individual;* pay no attention to the name of the disease. Many a doctor will prescribe carefully and hopefully for a patient suffering from a disease generally considered curable, but when treating a patient who has a disease *called incurable*, the same doctor will become demoralized, will prescribe carelessly, believing "there is no use trying."

The question at issue here is not one of dose, of quantity, but a question of law and principle *versus* haphazard carelessness. On the one side are men who try to relieve and to cure all cases of sickness; on the other, we find men who palliate their cases, giving a present temporary relief with a permanent aggravation later.—Editors.

